# RETRACTION: Antiresistin Neutralizing Antibody Alleviates Doxorubicin‐Induced Cardiac Injury in Mice

**DOI:** 10.1155/dim/9836978

**Published:** 2026-02-18

**Authors:** Disease Markers

RETRACTION: Y. Hu, J. Wang, W. Du, et al., “Antiresistin Neutralizing Antibody Alleviates Doxorubicin‐Induced Cardiac Injury in Mice,” *Disease Markers*, 2022, 3040521, https://doi.org/10.1155/2022/3040521.

The above article, published online on 13 December 2022 in Wiley Online Library (wileyonlinelibrary.com), has been retracted by agreement by John Wiley & Sons Ltd. The retraction has been agreed due to errors in the figures highlighted by the authors, and additional errors in the statistical analyses following further investigation. Specifically:

• The GAPDH blots in Figure 4a are identical to the t‐p65 blots shown in Figure 5d.
• In the statistical analyses, two‐way ANOVA should have been used followed by two group comparison.

In light of the above, the results and conclusions are no longer deemed reliable and the article is retracted.

The authors disagree with the retraction.

